# Generalization of the optical theorem: experimental proof for radially polarized beams

**DOI:** 10.1038/s41377-018-0025-x

**Published:** 2018-07-18

**Authors:** Alexey V. Krasavin, Paulina Segovia, Rostyslav Dubrovka, Nicolas Olivier, Gregory A. Wurtz, Pavel Ginzburg, Anatoly V. Zayats

**Affiliations:** 1 Department of Physics, King’s College London, Strand, London, WC2R 2LS UK; 2 Department of Electronic Engineering, Queen Mary University of London, London, UK; 3 School of Electrical Engineering, Tel Aviv University, Ramat Aviv, Tel Aviv 69978 Israel; 4 ITMO University, St. Petersburg, 197101 Russia; 5 Present Address: Departamento de Optica, CICESE, Carretera Ensenada-Tijuana, 3918, 22860 Ensenada Mexico; 6 Present Address: Laboratory for Optics & Biosciences, Ecole Polytechnique, CNRS, UMR 7645, F-91128 Palaiseau France; 7 Present Address: Department of Physics, University of North Florida, Jacksonville, FL 32224 USA

## Abstract

The optical theorem, which is a consequence of the energy conservation in scattering processes, directly relates the forward scattering amplitude to the extinction cross-section of the object. Originally derived for planar scalar waves, it neglects the complex structure of the focused beams and the vectorial nature of the electromagnetic field. On the other hand, radially or azimuthally polarized fields and various vortex beams, essential in modern photonic technologies, possess a prominent vectorial field structure. Here, we experimentally demonstrate a complete violation of the commonly used form of the optical theorem for radially polarized beams at both visible and microwave frequencies. We show that a plasmonic particle illuminated by such a beam exhibits strong extinction, while the scattering in the forward direction is zero. The generalized formulation of the optical theorem provides agreement with the observed results. The reported effect is vital for the understanding and design of the interaction of complex vector beams carrying longitudinal field components with subwavelength objects important in imaging, communications, nanoparticle manipulation, and detection, as well as metrology.

## Scattering: New light on optical theory

Conventional understanding of the scattering behavior of light and other electromagnetic radiation is challenged by studying beams with a complex structure, for example having a radial polarization. In radially polarized beams, the directions of the oscillating electric fields possess an axial symmetry, having components directed towards the center of the beam and along the beam direction. Researchers led by Alexey Krasavin at King’s College London, find that radially polarized beams of visible light and microwave radiation violate a common textbook description of light scattering, summarized in a mathematical relationship called the optical theorem. The researchers find that a revised generalized version of the optical theorem does, however, agree with the results they observe. They suggest this new understanding of complex electromagnetic beams should be useful for many applications, including imaging, communications, nanoparticle manipulation, detectors and metrology.

## Introduction

The optical theorem is a fundamental relation that emerges in both classical and quantum wave scattering phenomena^1^. It relates the amplitude of a wave scattered from an object in the forward direction to its extinction cross-section. However, the rigorous proof of the commonly used form of the theorem strongly relies on the scalar nature of the considered waves^1^ and can be easily extended only to a simple vectorial case of transverse plane waves^2–4^. While the former is usually true for acoustic or electron scattering (though even in the latter case, only for plane waves^5^), for electromagnetic waves both the spatial distribution of the incident field and its polarization structure can be important. It has been shown that tightly focused Gaussian beams scattered by a small spherical particle partially violate the optical theorem^6,7^. This violation is a direct consequence of the appearance of the longitudinal field components at the focal spot where the scattering object is located. The interaction of other beams of vectorial nature, such as radially or azimuthally polarized beams or complex vortex beams carrying optical angular momentum, with various nanostructures was studied both theoretically and experimentally^8–11^, but the dynamics of the scattering of such beams and its relationship to the fundamental properties of the optical theorem have not been considered. Meanwhile, electromagnetic beams with complex vectorial fields are currently used in many important applications, including high-resolution imaging, radar detection, communication technology, nanoparticle trapping and manipulation, surface metrology and many others for which the optical theorem is widely used in system design and performance evaluation.

Many versions of the optical theorem have been formulated for scenarios involving different geometries of illumination for which the textbook form of the theorem cannot be applied. For example, the application of the optical theorem to the scattering in transmission lines has been studied^12^, and generalizations for (i) nonlinear, time-varying and lossy materials^13^, (ii) anisotropic embedding media^14^, (iii) general inhomogeneous media^15^, (iv) surface waves and layered media^16^, and (v) evanescent fields^17^ have been theoretically derived. Nevertheless, in the majority of experimental studies where partial information regarding the scattering can be acquired, the optical theorem still tends to be applied in its conventional form.

In the present work, we experimentally demonstrate a complete violation of the conventional formulation of the optical theorem for vectorial beams and provide an experimental proof of its appropriate generalized version. Exploiting the vectorial structure of radially polarized beams in both optical and microwave spectral ranges, we study angular spectra of the scattering from subwavelength particles and show that scattering in the forward direction, which is favorable for the linearly polarized light illumination, vanishes under the radially polarized excitation even though the particle has a significant non-zero extinction cross-section. Therefore, the use of the conventional form of the optical theorem that establishes a direct relation between these two quantities is inappropriate. The violation of this connection was further confirmed in experiments for microwave radiation scattering through the mapping of the amplitude and phase of the forward scattering with a sub-diffraction resolution. As in the optical regime, the experimental results confirmed by numerical simulations, reveal strong overall scattering of the radially polarized beam with no forward scattering detected, in violation of the textbook optical theorem. Finally, the experimental and numerical results confirm the predictions based on the recent generalization of the optical theorem formulation.

## Materials and methods

### Finite element numerical modeling

Numerical analysis was performed using finite element software (COMSOL Multiphysics). The scattering from a 100 nm spherical gold nanoparticle (for which the permittivity data were taken from ref.^18^ was studied in the scattering-field formulation, with the simulation domain surrounded by a perfectly matched layer in order to guarantee the absence of back-reflection from the outer domain boundaries. The symmetry of the simulation setup (both the incident field and the object) was used to decrease the computational complexity. The simulation domain, which initially had a spherical form, was cut by two perpendicular planes intersecting along the beam axis. In the case of the plane wave illumination, these planes were the plane of the linear polarization and the wave vector plane perpendicular to it. For radial polarization, the azimuthal orientation of the planes is invariant due to the symmetry of the beam/object system. Only one quadrant of the space between these planes was used for numerical evaluation with the appropriate boundary conditions set on its flat edges (perfect magnetic conductor (PMC) for the polarization plane and perfect electric conductor (PEC) for the perpendicular plane in the case of linear polarization and PMC for both planes in the case of the radially polarized beam). The linearly polarized wave illumination was implemented in a straightforward manner, while the focused radially polarized beam field distribution obtained in the paraxial approximation^19,20^, was corrected numerically. The latter approach allowed the implementation of highly focused beams with a numerical aperture of 0.4 in air, corresponding to the ~3500 nm beam waist in the studied 300–1000 nm wavelength range, and with a numerical aperture of 1.42 in immersion oil, corresponding to the 550–900 nm waist, which is wavelength-dependent due to tight focusing. The validity of the model was checked by benchmarking it against the results for scattering of a linearly polarized plane wave on a metallic nanoparticle from the literature^21^. For all illumination scenarios (linear, radial, and azimuthal polarizations), the near field in the vicinity of the particle was numerically evaluated, while the far field was determined from the near field using the Stratton–Chu approach^22^.

### Optical Fourier microscopy

Single-particle scattering experiments were performed in reflection using 100 nm gold colloidal nanoparticles. Dispersed nanoparticles were placed in oil between two microscope coverglasses in order to minimize the index mismatch. Single nanoparticle spectroscopy was performed using an inverted microscope in which the back focal plane of the objective is imaged onto a CCD camera, allowing direct imaging of the scattering pattern of a single nanoparticle. For illumination, incoherent white light from a tungsten-halogen lamp was filtered spatially with a 5 μm diameter aperture and spectrally with a bandpass filter (532 nm, Chroma), collimated and polarized linearly using a grid polarizer (Thorlabs). The polarization can be transformed into radial polarization using a polarization converter (Arcoptix, Switzerland). Linearly or radially polarized light was focused on the particle with a 1.49NA, ×100 objective (Nikon). A nanoparticle was collocated with the center of the beam using a piezostage. For detection, the back-scattered signal was collected by the same objective lens, and its back aperture was imaged on a CCD using a four-lens system. The final images were obtained by subtracting the background signal measured from the coverglass alone from the signal detected in the presence of the nanoparticle. The back-scattering geometry provides a much weaker background, allowing a better signal-to-noise ratio than that for the forward direction while still allowing the connection to the forward scattering behavior via numerical simulations.

### Microwave scanning microscopy

The microwave experiments emulate the optical setup and additionally provide direct measurement of amplitude and phase distributions of the forward scattered waves^23^. The experiments were performed at the frequency of 9.5 GHz (corresponding to the wavelength *λ*=3.2 cm). A 3.5 mm sphere made from stainless steel was used as a scatterer. Both linearly and radially polarized beams were generated by a widely used conical horn antenna with an aperture diameter of 50 mm attached to a cylindrical waveguide fed by a coaxial cable^24^. The polarization of the beam (either linear or radial) was achieved by exciting different modes of the waveguide. To obtain linear polarization, the antenna was fed by a standard coaxial waveguide connected to a vector network analyzer (VNA, Agilent PNA-L-N5230C), yielding the fundamental *H*_11_ mode excited in a cylindrical waveguide. The working frequency (9.5 GHz) was chosen such that no other linearly polarized mode can propagate in the waveguide of this size (WC94, inner diameter of 24 mm). To obtain radial polarization, the excitation of the first radial mode of the cylindrical waveguide, namely, the *E*_01_ mode, was used. Here, as well, the waveguide size restricts any other non-polarized cylindrical waveguide modes in the given frequency band. Since zero-order Bessel functions, which describe the radial field distributions of *H*_11_ and *E*_01_ modes, mimic a Gaussian distribution with at least 97% overlap in amplitude, the field distributions at the antenna aperture imitate linearly or radially polarized Gaussian beams (at least near the center of the aperture), which are radiated in the free space. The amplitude, phase and polarization profiles of the radiated beams were confirmed by amplitude-resolved and phase-resolved measurements at the distance of approximately 1.5 wavelengths from the antenna aperture. A 3.5 mm sphere made from stainless steel was placed at the distance of one wavelength from the antenna aperture along the beam axis. The measurements of the scattered field were performed in the near-field, recording both phase and amplitude information with a sub-diffraction resolution using a probe mounted on a planar near-field scanner (NSI Inc.) and connected to a VNA, which allows signal acquisition in the spectral range up to 100 GHz. Mechanical movement of the probe was controlled by the scanner. A calibrated NSI probe (open end of the rectangular waveguide), specifically designed to work in the X-band (8–12 GHz), was used for the measurements of the transverse linearly polarized field. On the other hand, a custom-made coaxial probe was used to measure the longitudinal *z*-component of the field. First, the amplitude and phase *x–y* maps of the total (incident and scattered) field were measured at the distance of *λ*/2 from the scatterer. Then, the amplitude and phase maps of the incident field (with the scatterer removed) were measured exactly at the same detection plane, again for both polarizations. Using these data, the amplitude and phase of the scattered field were determined via phase-resolved field subtraction.

## Results and discussion

### Conventional optical theorem and complex vectorial beams

**Fig. 1:**
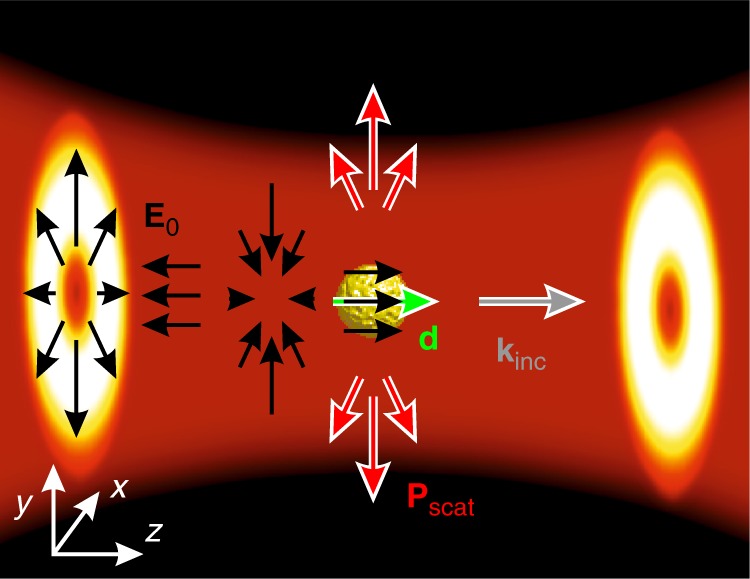
Schematic representation of a nanoparticle illuminated by a radially polarized beam. Evolution of the electric field structure of the incident beam illuminating the nanoparticle from the left (along **k**_inc_) is shown by black arrows together with the dipole moment (**d**) induced in the nanoparticle (green-white arrow).The intensity of the beam is shown by the color map. The directions of the power flow of the scattered field **P**_scat_ are shown by red-white arrows

The main contribution to the electromagnetic scattering from a subwavelength obstacle usually originates from a dipolar term in the multipolar decomposition. In other words, under linearly polarized plane wave illumination, the particle becomes predominantly polarized along the direction of the incident electrical field (if the magnetic response can be neglected) and re-radiates the energy according to the dipolar emission pattern. The highest intensity of the dipolar radiation propagates in the directions perpendicular to the dipole and, as the result, along the wave vector of the incident linearly polarized wave. Consequently, scattering in the forward direction (along the incident wave vector) is significant. The common formulation of the optical theorem postulates a direct proportionality between the scattering amplitude in the forward direction and the extinction cross-section^1–4^. However, the situation can be drastically different for vectorial beams that may carry longitudinal field components, optical angular momentum or transverse spin^25^. For example, radially polarized beams have a doughnut-like intensity profile for the transverse polarization directions and, most prominently, a strong longitudinal polarization component along the propagation direction at the beam axis^26^. The intensity map and the electric field structure of a focused radially polarized beam, the scattering of which will be studied below, are presented in Fig. 1.

The optical theorem in its textbook formulation provides a straightforward way to calculate the nanoparticle extinction cross-section ($C_{{\rm{ext}}}^{{\rm{OT}}}$) by relating its magnitude to the value of the far-field component of the normalized scattered electric field amplitude evaluated in the forward direction (along the incident wave vector) ${\mathbf{e}}_{{\rm{scat}}}^{{\rm{far}}}\left( {{\mathbf{k}} = {\mathbf{k}}_{{\rm{inc}}}} \right)$ ^2–4^:

1 $$C_{{\rm{ext}}}^{{\rm{OT}}} = \frac{{4\pi }}{{k\varepsilon _{\mathrm{d}}^{1/2}}}{\Im} \left\{ {{\mathbf{p}}^ \ast \cdot {\mathbf{e}}_{{\rm{scat}}}^{{\rm{far}}}\left( {{\mathbf{k}} = {\mathbf{k}}_{{\rm{inc}}}} \right)} \right\}$$

where **p** is the unit vector signifying the polarization of the incident wave, **k**_inc_ and **k** are the wave vectors of the incident and scattered waves, respectively,$\left| {\mathbf{k}} \right| = \left| {{\mathbf{k}}_{{\rm{inc}}}} \right| = 2\pi \varepsilon _{\mathrm{d}}^{1/2}/\lambda \,\epsilon_{d}$, is the permittivity of the surrounding dielectric, and ${\mathbf{e}}_{{\rm{scat}}}^{{\rm{far}}}\left( {{\mathbf{k}} = {\mathbf{k}}_{{\rm{inc}}}} \right)$ is related to the scattered electric field ${\mathbf{E}}_{{\rm{scat}}}^{{\rm{far}}}\left( {\mathbf{r}} \right)$ as follows:

2 $${\mathbf{E}}_{{\rm{scat}}}^{{\rm{far}}}\left( {\mathbf{r}} \right) = \left| {{\mathbf{E}}_0} \right|\frac{{e^{{ikr}}}}{r}{\mathbf{e}}_{{\rm{scat}}}^{{\rm{far}}}\left( {{\mathbf{k}},{\mathbf{k}}_{{\rm{inc}}}} \right)$$

where $\left| {{\mathbf{E}}_0} \right|$ is the amplitude of the incident wave.

Alternatively, the extinction cross-section can be evaluated directly,

3 $$C_{{\rm{ext}}}^{{\rm{dir}}} = C_{{\rm{abs}}}^{{\rm{dir}}} + C_{{\rm{scat}}}^{{\rm{dir}}}$$

as the sum of the absorption $C_{{\rm{abs}}}^{{\rm{dir}}}$ and scattering $C_{{\rm{scat}}}^{{\rm{dir}}}$ cross-sections. The absorption cross-section can be calculated as an integral of the absorption losses over the nanoparticle volume *V*, normalized to the incident power flow:

4 $$C_{{\rm{abs}}}^{{\rm{dir}}} = - \frac{{\mathop {\int}\limits_V {\frac{1}{2}{\Re} \left\{ {i\omega {\mathbf{E}} \cdot {\mathbf{D}}^ \ast } \right\}{\mathrm{d}}^3{\mathbf{r}}} }}{{\frac{{\varepsilon _{\mathrm{d}}\varepsilon _0c}}{2}\left| {{\mathbf{E}}_0} \right|^2}}$$

where **E**_0_ and **E** are the incident and total electric fields, respectively, and **D** is the electric displacement. The scattering cross-section can be calculated as an integral of the intensity of the scattered fields over a surface *S* enclosing the particle, normalized to the same incident field intensity:

5 $$C_{{\rm{scat}}}^{{\rm{dir}}} = \frac{{{\int} {\mathop {\int}\limits_S {{\mathbf{P}}_{{\rm{scat}}}^{{\rm{far}}}{\mathrm{d}}s} } }}{{\frac{{\varepsilon _{\mathrm{d}}\varepsilon _0c}}{2}\left| {{\mathbf{E}}_0} \right|^2}}$$

where ${\mathbf{P}}_{{\rm{scat}}}^{{\rm{far}}}$ is the power flow of the scattered waves. We note that other semi-analytical approaches, such as multipole expansion, can also be applied for calculations of extinction cross-sections for linearly or radially polarized beams^19^.

**Fig. 2:**
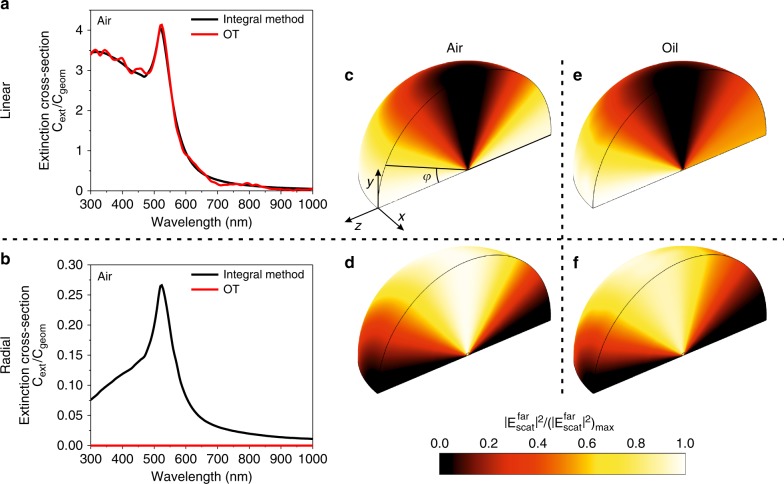
Breakdown of the conventional formulation of the optical theorem for a radially polarized beam. **a**, **b** Extinction cross-section spectra of gold nanoparticles with a radius of 50 nm (the data are normalized to the geometrical cross-section of the nanoparticle *C*_geom_), calculated using the optical theorem (Eq. (1), solid red lines) and by the direct evaluation of the sum of the absorption and scattering cross-sections (Eq. (3), black lines) for (**a**) linearly and (**b**) focused radially polarized illumination. **c**–**f** Angular scattering diagrams for the nanoparticle illuminated by (**c**, **e**) a plane wave linearly polarized along the *y*-direction and (**d**, **f**) a focused radially polarized beam. The illuminating wave propagates along the *z*-direction and has the wavelength *λ* = 530 nm. The particle is located at the center of the diagram

For the plane wave illumination of a gold nanoparticle, the direct method for the calculation of the extinction cross-section ($C_{{\rm{ext}}}^{{\rm{dir}}}$) and the method based on the optical theorem ($C_{{\rm{ext}}}^{{\rm{OT}}}$) show excellent agreement (Fig. 2a). The extinction in the spectral range *λ* = 480–550 nm corresponds to the plasmonic dipolar resonance of the particle. The angular distribution of the far-field scattering has the characteristic shape corresponding to a dipolar radiation pattern, namely, cos^2^*φ*, where *φ* is the scattering angle (Fig. 2c). As expected, the scattering dipole is induced along the *y*-direction along the polarization of the incident plane wave.

The situation is drastically different for the radially polarized incident beam. The wavelength dependence of the extinction cross-section calculated using the direct integration method shows a distinctive peak at the localized surface plasmon resonance of the particle (Fig. 2b, black line), which is in good agreement with the case of the plane wave excitation (Fig. 2a). At the same time, the extinction cross-section evaluated using the optical theorem given by Eq. (1) is practically zero (Fig. 2b, red line), within a numerical noise defined by the accuracy of the simulations. This means that the optical theorem in its common form cannot be applied for such beams. Here, we note that for the case of optical beams focused to dimensions comparable to the size of the scattering object, reconsideration of the usual notion of the cross-section is required due to the variation of the beam intensity across the object. For example, in the case of scattering of localized electron wave packets, this was done via the definition of the cross-section through the number of the scattering events, normalized to the introduced effective luminosity of the wave packet^5^. In our case, in analogy to this approach, we used the power extinguished from the beam (equivalent to the number of photons removed from the beam), normalizing it for simplicity to the maximum intensity in the focal plane (Fig. 1).

The apparent contradiction created by the optical theorem described by Eq. (1) can be understood from the angular scattering diagram (Fig. 2d). For the radially polarized beam, the entire nanoparticle is located in the region of space where the longitudinal component of the incident field dominates (Fig. 1) and the transverse polarization is vanishingly small. Furthermore, it has an axisymmetric structure with the transverse fields directed out of the beam axis at the location of the scatterer, prohibiting the excitation of a transverse dipolar mode (Fig. 1). Consequently, the dipole moment in the particle is excited only by the longitudinal components along the beam axis, giving rise to pronounced energy re-radiation directed perpendicular to the latter. The dipolar excitation also causes the related absorption losses in the metal particle. This leads to significant non-zero values of scattering and absorption cross-sections, resulting in a considerable value of the extinction cross-section. However, the optical theorem given by Eq. (1) fails to reflect this: due to the symmetry, the re-radiation of the longitudinal dipole in the forward direction (along the dipole axis) is zero, and therefore, the optical theorem returns a zero extinction cross-section (Fig. 2d). The visual comparison between Fig. 2c and d suggests that they are almost perfect replicas of each other if a 90° rotational transformation is applied to either one of them. This observation enables us to draw an intuitive conclusion regarding the source of the violation of the optical theorem in this formulation. Instead of traveling along the optical axis, the scattering is deflected by 90°, which is the optimal angle for minimizing the forward scattering. Moreover, as seen in Fig. 2e, f, the violation also occurs in the case of a nonresonant excitation at *λ* = 520 nm for the nanoparticle in oil (the resonance is moved in this case to *λ* = 600 nm), which was experimentally examined in further studies described in the next section. In the case of the focused Gaussian beam, both transverse (dominant) and longitudinal (which are the consequence of focusing) components are present at the particle position. As was shown above, for the latter components, there is a complete violation of the optical theorem, while for the former components, the optical theorem holds, overall leading to a partial theorem violation. Generally, stronger beam focusing corresponds to a higher ratio between the longitudinal and transverse components and a more significant violation of the optical theorem. As was found in ref.^5^ for the axisymmetric scattering of vortex electron wave packets, zero forward scattering and yet a non-zero overall scattering cross-section can be observed even for scalar localized incident fields of a complex structure with a vortex phase change around the wave packet axis. However, the vectorial nature of the electromagnetic field essentially amplifies the effect: the zero divergence of the electromagnetic field at the node of the fields at the beam axis leads to the presence of the longitudinal field components, resulting in efficient excitation of the dipolar mode in the nanoparticle along the beam axis and thus efficient scattering, resulting in essential extinction simultaneously with zero forward scattering.

### Experimental investigation of scattering in the optical domain using Fourier microscopy

**Fig. 3:**
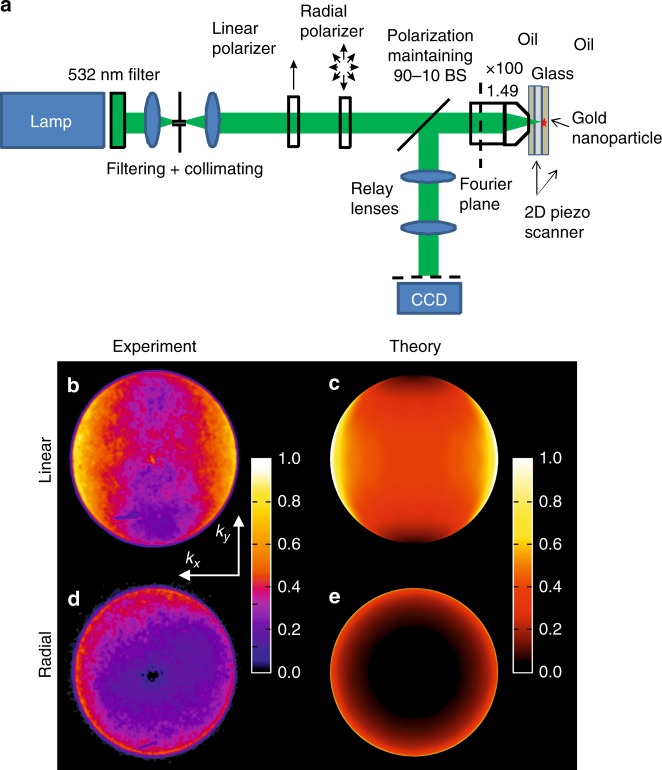
**Experiments in the visible spectral range. a** Schematic of the Fourier imaging setup used in the experiments. **b**–**e** Angular distribution of the far-field back-scattering in the case of a nanoparticle illuminated by (**b**, **c**) a linearly polarized plane wave and (**d**, **e**) a radially polarized beam. The results obtained in the optical experiments (**b**, **d**) are compared with the finite element method numerical modeling (**c**, **e**). The parameters of the nanoparticle and illumination are as in Fig. 2

To verify the predictions of the numerical modeling, single-particle spectroscopy was performed, allowing the direct imaging of the scattering pattern (Materials and methods section) (Figure 3a). First, linearly polarized excitation was studied with a flat linearly polarized wavefront at the center of the focal spot. These excitation conditions can be directly compared to a plane wave excitation implemented in the simulations. Figure 3b, c show excellent agreement of the experimental and theoretical angular distribution in the back-scattering zone, clearly demonstrating a wave vector distribution that is symmetric with respect to the *k*_*y*_ = 0 plane with gradually decreasing magnitude toward higher *k*_*y*_. They directly demonstrate the excitation of a dipole along the excitation polarization direction, as seen from the field map cross-sections of the full scattering diagram (Fig. 2e). Due to the nature of the Fourier plane detection, the number of the wave vectors per (*dk*_*x*_, *dk*_*y*_) interval is recorded. Hence, for a uniform angular distribution, the wave vectors scattered at higher angles have higher density than those close to the optical axis. This explains the higher intensities at the sides of the Fourier images in Fig. 3b, c for higher *k*_*x*_.

For radial polarization, the experimental and numerical observations are again in excellent agreement (cf. Fig. 3d, e). It can be seen that the Fourier intensity in the entire central region of the map around *k*_*x*_ = *k*_*y*_ = 0 is virtually zero, gradually increasing toward higher *k*_*x*_ and *k*_*y*_ and clearly possessing a polar angular symmetry. Since the subwavelength size of the sphere only allows the dipolar plasmonic resonance, this provides clear evidence that the direction of the excited dipole is along the *z*-axis, which can be easily seen by the comparison of wave vector distributions in the Fourier images with the full scattering diagram in Fig. 2f. Such an orientation of the excited dipole inevitably leads to the absolute zero value of the scattering field (and consequently its imaginary part) in the forward direction along the *z*-axis. Hence, we demonstrated a complete violation of the optical theorem in its conventional form for radially polarized beams both experimentally and numerically: while the optical theorem predicts an extinction cross-section to be zero $C_{{\rm{ext}}}^{{\rm{OT}}} = 0$ (Eq. (1)) on the basis of the zero scattering along the incident wave vector, a considerable extinction cross-section of the particle is observed with strong scattering of the incident radially polarized beam in the direction of large *k*_*x*_ and *k*_*y*_ wave vectors.

### Scanning microscopy of a scattered field in the microwave domain

**Fig. 4:**
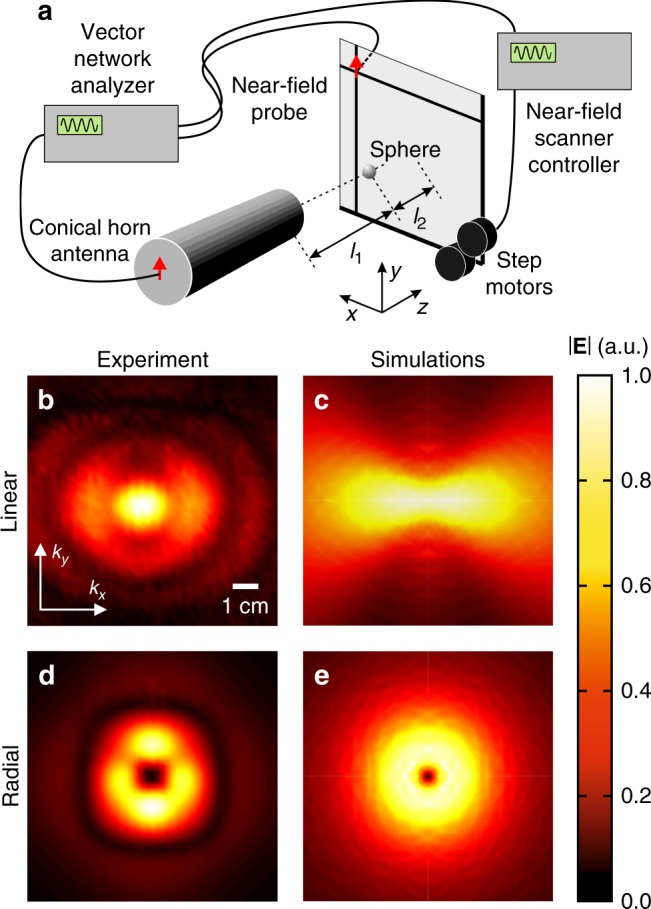
Experiments in the microwave spectral range. **a** Near-field scanning setup used in the microwave experiments. **b**–**e** Near-field distribution of forward scattering on a 3.5 mm metallic nanoparticle recalculated from the amplitude and phase maps measured at a distance of *l*_2_ = 14.5 mm from the particle center in the case of (**b**, **c**) a linearly polarized plane wave and (**d**, **e**) a radially polarized beam. The microwave radiation frequency is 9.5 GHz, corresponding to *λ* = 3.2 cm ($l_1 \approx \lambda$). The results obtained in the microwave experiments (**b**, **d**) are compared with the finite element method numerical modeling (**c**, **e**)

The microwave scattering experiments emulate the optical setup and provide direct measurements of the amplitude and phase distributions of the scattered waves^23^. The distributions of the scattered field amplitude in the forward direction demonstrate very good agreement with the modeling results (cf. Fig. 4b–e). For the linearly polarized illumination, the radiation profile corresponds to the dipolar moment excited along the direction of the incident polarization (Fig. 4b). On the other hand, the radiation profile in the case of the radially polarized illumination provides unambiguous evidence for the dipole moment excitation in the *z*-direction, along the beam axis (Fig. 4d). Again, the conventional formulation of the optical theorem is violated in this case: an essential scattering signal (and, therefore, considerable extinction cross-section) is evident, while Eq. (1) predicts zero extinction on the basis of the zero field measured in the forward direction (along the *z*-axis).

The fact that the facet of the probe rectangular waveguide lies in the measurement plane and, therefore, has a lower acceptance of the incident wave at higher angles leads to the smaller measured signal in this region, explaining the related difference between the experimental and theoretical field maps (cf. Fig. 4b with d and Fig. 4c with e). Also, the metallic sphere was held in the setup by a sample holder made of a plastic foam sheet. The sheet is nearly transparent for microwaves (*ε*~1.1) and, thus, did not essentially influence the results, although its presence can explain minor radial interference fringes observed in the experimental field maps (Fig. 4b, d).

### Generalization of the optical theorem

These numerical and experimental observations provide strong motivation for the development and application of generalized formulations of the optical theorem^27^. This can be achieved by considering a relation linking extinction to incident (**E**_0_(**r**), **H**_0_(**r**)) and total (**E**(**r**), **H**(**r**)) fields valid for any vectorial structure of the field^27^:

6 $$	C _{{\rm{ext}}}^{{\rm{GOT}}} = \frac{1}{{\varepsilon _{\mathrm{d}}\left| {{\mathbf{E}}_0} \right|^2}} \\ 	 {\Im}\left\{ {\mathop {\int}\limits_V {{\mathrm{d}}^3{\mathbf{r}}k\left( {\mathbf{r}} \right)\left[ {{\mathbf{E}}_0^ \ast \left( {\mathbf{r}} \right) \cdot {\mathbf{E}}\left( {\mathbf{r}} \right)\eta \left( {\mathbf{r}} \right) + {\mathbf{H}}_0^ \ast \left( {\mathbf{r}} \right) \cdot {\mathbf{H}}\left( {\mathbf{r}} \right)\chi \left( {\mathbf{r}} \right)} \right]} } \right\}$$

where *η*(**r**) and *χ*(**r**) are the electric and magnetic susceptibilities of the scatterer and the integration is taken over the scatterer volume *V*. The incident beam in free space is then represented by an arbitrary complex vectorial structure allowed by Maxwell’s equations and treated as a superposition of plane waves (generally also allowing the evanescent components) with amplitudes **e**(**k**). The extinction cross-section can then be expressed by projecting the amplitudes of the scattered components ${\mathbf{e}}\left( {{\mathbf{k}}_1} \right){\mathbf{A}}\left( {{\mathbf{k}}_1,{\mathbf{k}}_2^ \ast } \right)$ from each of the partial incident waves **e**(**k**_1_) on all other incident components **e**(**k**_2_) and integrating over all possible directional combinations of **k**_1_ and **k**_2_:

7 $$	C_{{\rm{ext}}}^{{\rm{GOT}}} = \frac{\omega }{{\varepsilon _{\mathrm{d}}c\left| {{\mathbf{E}}_0} \right|^2}} \\ 	{\Im}\left\{ {{\int} {{\mathrm{d}}^2k_{1||}{\int} {{\mathrm{d}}^2k_{2||}e_\beta ^ \ast \left( {{\mathbf{k}}_2} \right)e_\alpha \left( {{\mathbf{k}}_1} \right)A_{\alpha \beta }\left( {{\mathbf{k}}_1,{\mathbf{k}}_2^ \ast } \right)} } } \right\}$$

**Fig. 5:**
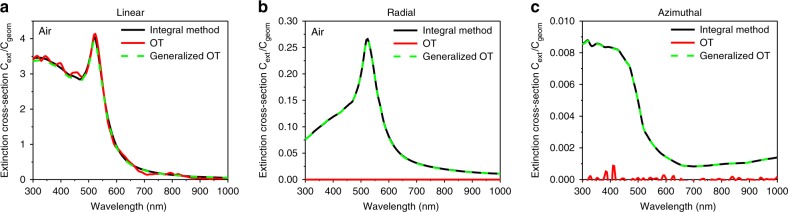
Comparison of the conventional and generalised formulations of the optical theorem and numerical modelling for linearly, radially and azimuthally polarized beams. Extinction cross-section spectra of gold nanoparticles with a radius of 50 nm, calculated using the optical theorem (OT) Eq. (1) (solid red line), generalized OT Eqs. (6, 7) (dashed green line) and direct evaluation of the sum of the absorption Eq. (4) and scattering Eq. (5) cross-sections (black line) for (**a**) linearly, (**b**) radially and (**c**) azimuthaly polarized beams

Here, $A_{\alpha \beta }\left( {{\mathbf{k}}_1,{\mathbf{k}}_2^ \ast } \right)$ are the components of the scattering amplitude tensor ${\mathbf{A}}\left( {{\mathbf{k}}_1,{\mathbf{k}}_2^ \ast } \right)$, double indices imply summation and || denotes the projection on the *z*-axis. Applying this approach to the scattering of both linearly and radially polarized light, it was found that the generalized optical theorem (Eqs. (6) and (7)) provides results in excellent agreement with the direct evaluation of the extinction cross-section using Eqs. (3–5) (Fig. 5a, b), revealing its validity even for the vectorial case. We note that in the derivation of the generalized optical theorem, no assumptions regarding the shape of the incident beam and the scattering object were made, e.g., complex fields including evanescent components and objects with an optical response dominated by magnetic dipoles or electric quadrupoles can be considered. The form of the optical theorem given by Eq. (6) is very convenient for approaching the problem with numerical simulations, allowing straightforward integration of the resulting electromagnetic fields. On the other hand, the form given by Eq. (7) is more suitable for analytical and semi-analytical calculations. Finding ${\mathbf{A}}\left( {{\mathbf{k}}_1,{\mathbf{k}}_2^ \ast } \right)$ and evaluating the scattering of a plane wave by an object of a given shape are generally within the capabilities of analytical calculations^28^. Then, the double integration over the wave vectors for simple scatterers can be done analytically; otherwise, numerical evaluation can be performed using standard software.

The optical theorem was further tested in the case of the scattering of a focused azimuthally polarized beam (with the same parameters as for the radially polarized beam in Fig. 2) on a 100 nm spherical gold nanoparticle. Numerical modeling shows that the generalized version of the optical theorem (Eqs. (6) and (7), dashed green line in Fig. 5c) provides the correct prediction of the value of the extinction cross-section, while the conventional formulation (Eq. (1), solid red line in Fig. 5c) proves to be inadequate. Due to the vectorial structure of the beam signified by the absence of the electric terms in its multipole decomposition^29^, the electric resonances that play a leading role in the optical response of a spherical plasmonic particle are not excited. This leads to much lower extinction cross-section values compared to linearly and radially polarized excitations (cf. Fig. 5c and Fig. 5a, b). In particular, the peak at the wavelength of 520 nm corresponding to the dipole resonance is no longer present for the azimuthal polarization. The observed small increase in the extinction cross-section toward the shorter wavelengths is related to the increase of the absorption in the metal.

## Conclusions

A violation of the standard formulation of the optical theorem was demonstrated both experimentally and numerically for illumination with radially polarized electromagnetic beams featuring strong longitudinal field components. Optical measurements were complemented by experiments in the microwave domain. The experiments together with comprehensive numerical modeling show clear evidence of the breakdown of the textbook version of the optical theorem. The origin of the breakdown has been identified to be mainly due to the presence of longitudinal field components in the complex vector beams. Rather than creating a paradox, this violation provides the evidence of the need for the reconsideration of the conditions required for satisfying this theorem, which was originally formulated for scalar and transverse vectorial waves. The generalized formulation of the optical theorem^27^ was shown to be in agreement with our numerical and experimental results. Longitudinal field components and related transverse optical spin of the surface and guided modes also lead to an inverse photonic spin-Hall effect, which is impossible for transverse waves^30^, and the emergence of lateral optical forces^31^. Finally, we would like to note that a similar violation should be expected for another important type of complex beams – vortex beams (with a non-zero orbital angular momentum). In analogy to radially and azimuthally polarized beams, vortex beams have vortex phase changes around their axes. Due to the symmetry of the problem, the forward scattering for vortex beams is zero, and so is the extinction cross-section given by the standard expression for the optical theorem, while, in fact, the extinction cross-section is non-zero at least due the high-order (e.g., quadrupolar) scattering effects and, if present, due to the absorption.

To underline the importance of the generalization of the well-established physical rules to novel phenomena for which the original form of the rule is no longer applicable, it is interesting to draw the attention to an example having very close parallels to the effects studied in the manuscript, yet from a completely different area of physics. Specifically, this is the generalization of the Born approximation for the scattering of electrons on energy potentials in the case of localized electron wave packets instead of plane waves^5^^.^^32^, Apart from reconsidering the definition of the cross-section when the incident field localization is comparable to the scatterer size, it was found that for twisted (vortex) incident electron wave packets, if the impact parameter of the incident wave packet is zero (the beam axis goes through the center of the axisymmetric scattering potential), the forward scattering is zero as well, while the overall cross-section is not, which is in complete analogy to the violation of the optical theorem for the radial and azimuthal optical beams considered here.

Since the optical theorem in different forms is frequently used in a broad range of applications, such as imaging, nanoparticle manipulation, communications, and radar detection, as well as in other areas of physics, e.g., for quantum scattering^33^, these findings demonstrate that a careful reconsideration of the required conditions and the introduction of additional degrees of freedom are of key importance. The understanding and generalization of the optical theorem applicability can broaden the span of its applications. For example, consideration of the spin-flip in an electron scattering process could introduce another (vectorial) degree of freedom, which could have remarkable implications for the relationships between the total extinction and forward scattering.

## Data availability

All data supporting this research are provided in full in the results section.

## References

[1] Born M, Wolf E (1999). Principles of Optics.

[2] Jackson JD (1999). Classical Electrodynamics.

[3] Bohren CF, Huffman DR (1998). Absorption and Scattering of Light by Small Particles.

[4] Mishchenko MI, Travis LD, Lacis AA (2002). Scattering, Absorption, and Emission of Light by Small Particles.

[5] Karlovets DV, Kotkin GL, Serbo VG, Surzhykov A (2017). Scattering of twisted electron wave packets by atoms in the Born approximation. Phys. Rev. A.

[6] Newton RG (1976). Optical theorem and beyond. Am. J. Phys..

[7] Lock JA, Hodges JT, Gouesbet G (1995). Failure of the optical theorem for Gaussian-beam scattering by a spherical particle. J. Opt. Soc. Am. A.

[8] Şendur K, Şahinöz A (2009). Interaction of radially polarized focused light with a prolate spheroidal nanoparticle. Opt. Express.

[9] Normatov A (2010). Efficient coupling and field enhancement for the nano-scale: plasmonic needle. Opt. Express.

[10] Lerman GM, Yanai A, Levy U (2009). Demonstration of nanofocusing by the use of plasmonic lens illuminated with radially polarized light. Nano. Lett..

[11] Chen WB, Abeysinghe DC, Nelson RL, Zhan QW (2009). Plasmonic lens made of multiple concentric metallic rings under radially polarized illumination. Nano. Lett..

[12] Marengo EA, Tu J (2014). Optical theorem for transmission lines. Prog. Electromagn. Res. B.

[13] Marengo EA, Tu J (2016). Generalized optical theorem in the time domain. Prog. Electromagn. Res. B.

[14] Marengo EA (2013). A new theory of the generalized optical theorem in anisotropic media. IEEE Trans. Antennas Propagat.

[15] Dacol DK, Roy DG (2005). Generalized optical theorem for scattering in inhomogeneous media. Phys. Rev. E.

[16] Halliday D, Curtis A (2008). Generalized optical theorem for surface waves and layered media. Phys. Rev. E.

[17] Carney PS (1999). The optical cross-section theorem with incident fields containing evanescent components. J. Mod. Opt..

[18] Johnson PB, Christy RW (1972). Optical constants of the noble metals. Phys. Rev. B.

[19] Mojarad NM, Agio M (2009). Tailoring the excitation of localized surface plasmon-polariton resonances by focusing radially-polarized beams. Opt. Express.

[20] Gouesbet G, Lock JA, Gréhan G (2011). Generalized Lorenz–Mie theories and description of electromagnetic arbitrary shaped beams: Localized approximations and localized beam models, a review. J. Quant. Spectrosc. Radiat. Transf..

[21] Parsons J, Burrows CP, Sambles JR, Barnes WL (2010). A comparison of techniques used to simulate the scattering of electromagnetic radiation by metallic nanostructures. J. Mod. Opt..

[22] Stratton JA, Chu LJ (1939). Diffraction theory of electromagnetic waves. Phys. Rev..

[23] Kapitanova PV (2014). Photonic spin Hall effect in hyperbolic metamaterials for polarization-controlled routing of subwavelength modes. Nat. Commun..

[24] Balanis CA (2005). Antenna Theory: Analysis and Design.

[25] Bliokh KY, Rodríguez-Fortuño FJ, Nori F, Zayats AV (2015). Spin–orbit interactions of light. Nat. Photonics.

[26] Martínez-Herrero R, Mejías PM, Juvells I, Carnicer A (2012). Transverse and longitudinal components of the propagating and evanescent waves associated to radially polarized nonparaxial fields. Appl. Phys. B.

[27] Lytle DR, Carney PS, Schotland JC, Wolf E (2005). Generalized optical theorem for reflection, transmission, and extinction of power for electromagnetic fields. Phys. Rev. E.

[28] Liou KN, Cai Q, Pollack JB, Cuzzi JN (1983). Light scattering by randomly oriented cubes and parallelepipeds. Appl. Opt..

[29] Orlov S, Peschel U, Bauer T, Banzer P (2012). Analytical expansion of highly focused vector beams into vector spherical harmonics and its application to Mie scattering. Phys. Rev. A.

[30] O’Connor D, Ginzburg P, Rodríguez-Fortuño FJ, Wurtz GA, Zayats AV (2014). Spin–orbit coupling in surface plasmon scattering by nanostructures. Nat. Commun..

[31] Rodríguez-Fortuño FJ, Engheta N, Martínez A, Zayats AV (2015). Lateral forces on circularly polarizable particles near a surface. Nat. Commun..

[32] Karlovets DV, Kotkin GL, Serbo VG (2015). Scattering of wave packets on atoms in the Born approximation. Phys. Rev. A.

[33] Taylor JR (2006). Scattering Theory: The Quantum Theory of Nonrelativistic Collisions.

